# Localization of Impaired Kinesthetic Processing Post-stroke

**DOI:** 10.3389/fnhum.2016.00505

**Published:** 2016-10-17

**Authors:** Jeffrey M. Kenzie, Jennifer A. Semrau, Sonja E. Findlater, Amy Y. Yu, Jamsheed A. Desai, Troy M. Herter, Michael D. Hill, Stephen H. Scott, Sean P. Dukelow

**Affiliations:** ^1^Division of Physical Medicine and Rehabilitation, Department of Clinical Neurosciences, Hotchkiss Brain Institute, University of CalgaryCalgary, AB, Canada; ^2^Calgary Stroke Program, Department of Clinical Neurosciences, University of CalgaryAB, Canada; ^3^Department of Exercise Science, University of South CarolinaColumbia, SC, USA; ^4^Department of Biomedical and Molecular Sciences, Queen's UniversityKingston, ON, Canada

**Keywords:** kinesthesia, proprioception, voxel lesion symptom mapping (VLSM), robotics, sensory impairment, stroke

## Abstract

Kinesthesia is our sense of limb motion, and allows us to gauge the speed, direction, and amplitude of our movements. Over half of stroke survivors have significant impairments in kinesthesia, which leads to greatly reduced recovery and function in everyday activities. Despite the high reported incidence of kinesthetic deficits after stroke, very little is known about how damage beyond just primary somatosensory areas affects kinesthesia. Stroke provides an ideal model to examine structure-function relationships specific to kinesthetic processing, by comparing lesion location with behavioral impairment. To examine this relationship, we performed voxel-based lesion-symptom mapping and statistical region of interest analyses on a large sample of sub-acute stroke subjects (*N* = 142) and compared kinesthetic performance with stroke lesion location. Subjects with first unilateral, ischemic stroke underwent neuroimaging and a comprehensive robotic kinesthetic assessment (~9 days post-stroke). The robotic exoskeleton measured subjects' ability to perform a kinesthetic mirror-matching task of the upper limbs without vision. The robot moved the stroke-affected arm and subjects' mirror-matched the movement with the unaffected arm. We found that lesions both within and outside primary somatosensory cortex were associated with significant kinesthetic impairments. Further, sub-components of kinesthesia were associated with different lesion locations. Impairments in speed perception were primarily associated with lesions to the right post-central and supramarginal gyri whereas impairments in amplitude of movement perception were primarily associated with lesions in the right pre-central gyrus, anterior insula, and superior temporal gyrus. Impairments in perception of movement direction were associated with lesions to bilateral post-central and supramarginal gyri, right superior temporal gyrus and parietal operculum. All measures of impairment shared a common association with damage to the right supramarginal gyrus. These results suggest that processing of kinesthetic information occurs beyond traditional sensorimotor areas. Additionally, this dissociation between kinesthetic sub-components may indicate specialized processing in these brain areas that form a larger distributed network.

## Introduction

Proprioception is traditionally thought to be comprised of two components, position sense and movement sense (kinesthesia) (Sherrington, 1907; Goodwin et al., 1972). Humans have both static (position sensitive) and dynamic (movement sensitive) peripheral muscle receptors (Proske and Gandevia, 2012). Following stroke, kinesthetic impairments are associated with reduced functional independence (Torre et al., 2013). Impairments specific to kinesthesia after stroke have only recently been systematically quantified and are present in approximately two-thirds of stroke survivors (Semrau et al., 2013). Case series level evidence has demonstrated post-stroke brain lesion locations associated with “abnormal” position sense (including the thalamus, internal capsule, and post-central gyrus) (Kim, 1992, 2007; Tong et al., 2010). However, much less is known about the underlying neuroanatomy of impaired kinesthesia (Kenzie et al., 2014).

Functional neuroimaging studies in healthy individuals (Naito et al., 1999), using either vibration of muscle tendons to induce kinesthetic illusions or passive joint movements, have shown that the primary and secondary somatosensory cortices (Mima et al., 1999), primary motor cortex, premotor cortex (Weiller et al., 1996), supplementary motor area (Naito et al., 1999), inferior parietal lobule, superior temporal sulcus, and cerebellum are all associated with perceptions of joint movement (Romaiguère et al., 2003; Kavounoudias et al., 2008). Activation of subcortical structures such as the thalamus and basal ganglia (Naito et al., 2007; Goble et al., 2012) have also been reported. These studies do not describe how or if the brain independently processes specific aspects of kinesthesia such as speed, direction, and amplitude of limb movement, or the neuroanatomical structures required for this processing.

With a validated, quantitative measure of kinesthesia using a robotic exoskeleton (Semrau et al., 2013), we aimed to identify the lesion sites associated with specific aspects of impaired kinesthesia (speed, direction, and amplitude), since these impairments are often not identified clinically or well understood anatomically. Recent work in our lab has also shown that these kinesthetic impairments are significantly associated with functional independence post-stroke (Semrau et al., 2013). We examined a large sample of stroke survivors with cerebral lesions using voxel-based lesion symptom mapping (VLSM) (Bates et al., 2003), and a statistical region of interest (sROI) analysis. VLSM is a powerful method to analyze structure-function relationships in the brain on a voxel-by-voxel basis, since it does not require a priori assumptions about brain anatomic structure-function correlation. A large sample is used to determine whether damage at a given voxel is associated with behavioral impairment. This informs us which brain structures are associated with that given behavior. We employ a sROI analysis as a complementary method to VLSM, because it provides improved statistical power by reducing the number of comparisons being made.

Given what is currently known about the central processing of proprioception, we assessed the hypothesis that lesions to known somatosensory structures arising from the dorsal column-medial lemniscal pathway (ventral posterior lateral nucleus of the thalamus, posterior limb of the internal capsule, and post-central gyrus) would result in measureable kinesthetic impairments.

## Materials and methods

### Subjects

Subjects with sub-acute ischemic stroke (*n* = 142) were recruited from the Foothills Medical Centre or Dr. Vernon Fanning Centre in Calgary Alberta, Canada. All subjects were 18 years or older at the time of assessment. Subjects were excluded for the following reasons: clinical diagnosis of stroke prior to the current one, hemorrhagic stroke, stroke affecting both sides of the brain, stroke in the brainstem, no identifiable lesion on MRI or CT, pre-existing neurological disorder (i.e., diagnosis of Parkinson's Disease, Multiple Sclerosis, etc.), orthopedic problems, neuropathy, or pain in either upper extremity, or inability to follow the instructions for the robotic assessment due to aphasia, language barriers, apraxia, or cognitive deficits. Cerebellar strokes were excluded due to software limitations that resulted in poor normalization to the Montreal Neurological Institute (MNI) template brain. Subjects were also excluded if they demonstrated motor impairments of the ipsilesional arm on clinical assessment. Subjects were required to be alert while performing the task and were excluded if fatigue was determined to be limiting participation by the therapist operating the robot. All individuals provided written informed consent prior to study participation in accordance with the Declaration of Helsinki. This study was approved by the University of Calgary Research Ethics Board.

### Robotic assessment of kinesthesia

#### Robot set-up and kinesthesia task

**Figure 1:**
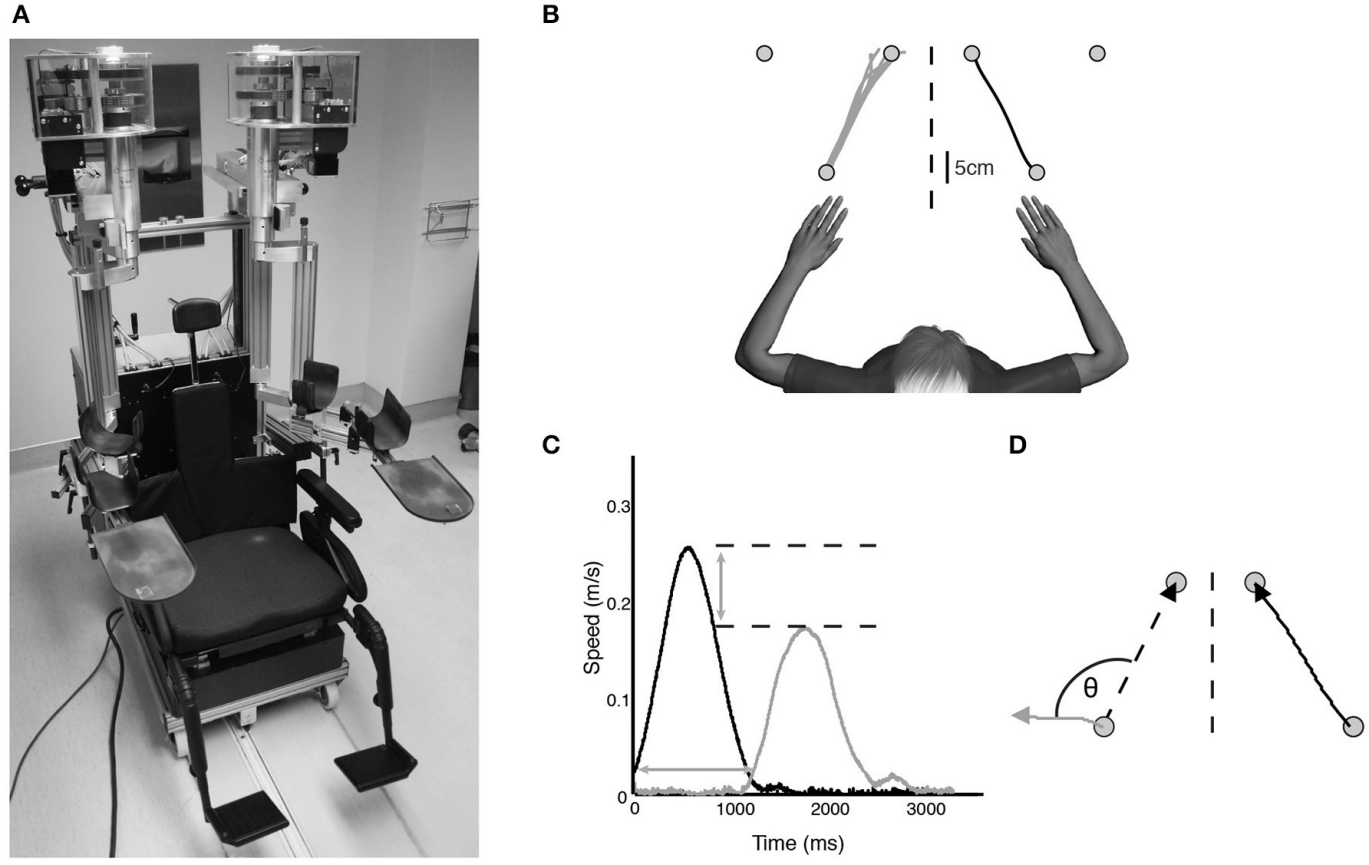
**(A)** KINARM exoskeleton (BKIN Technologies Ltd., Kingston, Ontario). Subjects sat in the wheelchair base with both arms supported against gravity by the arm troughs. Vision of the upper extremities was occluded with a bib fitted around the neck and the video display was occluded (not pictured). **(B)** Overhead view of exemplar control data for one movement direction of the kinesthesia task. The stroke-affected arm (black line) was moved by the robot and subjects mirror-matched the movement as soon as they felt the robot move (gray lines). **(C)** Speed profile for a single movement of a typical stroke subject. Black bell-shaped profile indicates passive arm movement driven by the robot, gray bell-shaped profile indicates subject movement. Time between passive and active arm movement onset indicates response latency. The difference in peak speed (horizontal dotted lines) between the passive arm and active arm represents peak speed ratio. **(D)** Spatial profile for a single movement of an exemplar stroke subject. Angular deviation between the ideal (left dotted line) and actual movement path (gray line) at peak hand speed indicates initial direction error. Path length ratio was calculated as the length of the active hand movement (gray line) divided by the length of the passive hand movement (black line).

A KINARM exoskeleton robot (BKIN Technologies Ltd., Kingston, ON) (Figure 1A) was used to assess kinesthesia of the upper extremities. This task (Semrau et al., 2013) takes ~5 min to complete. Briefly, individuals were seated in the wheelchair base with both arms supported against gravity by the robotic arms in a near frictionless environment and were free to move in the horizontal plane (Figure 1B). Each subject was fitted to the robotic exoskeleton by the study therapist or physician to ensure free movement of both arms and a centered body position. Vision of the upper extremities was occluded using a bib attached around the subject's neck and a blind over the subject's arms.

Prior to the start of each trial both hands were positioned (one by the robot, one actively by the subject) in mirrored locations in the workspace at one of three pre-determined locations (Figure 1B). This ensured the hands were at the same starting position before beginning each trial. After a brief delay the robot moved the stroke affected arm (passive arm) to one of the other target locations in a straight line with a bell-shaped velocity profile (peak speed = 0.28 m/s, distance = 20 cm) (Figure 1C). Subjects were required to use their unaffected arm (active arm) to mirror-match the speed, direction and magnitude of the robotically moved stroke affected arm. Subjects were instructed to move as soon as they felt the robot move their arm, mirror-matching the movement with the opposite arm in real time. All subjects with stroke generated active movements with their unaffected arm. Movements were performed six times in each of the six directions for a total of 36 trials.

#### Kinesthetic measurements

A total of eight movement parameters were measured from the robotic task. *Response Latency* (Figure 1C) was the time between the start of the robot movement and the start of the subject-initiated movement. The start of movement was defined as the point at which subjects exceeded 10% of maximum hand speed. *Peak speed ratio* (Figure 1C) was the ratio of peak hand speed of the passive and active arms. Ratios equal to 1 indicated perfect speed matching, whereas ratios less than or greater than 1 indicated movements slower or faster than passive arm speed, respectively. *Initial direction error* (Figure 1D) was the angular deviation between movement paths of the passive and active arms from movement onset to peak hand speed. *Path length ratio* (Figure 1D) was measured as the total path length of the active arm divided by the total path length of the passive arm. Ratios of 1 indicated perfect matching of active to passive movement length, with values less than or greater than 1 indicating shorter or longer movement lengths, respectively. Variability between trials on all of these parameters was measured as the standard deviation across the 36 trials. In total, the eight parameters were comprised of the means and standard deviations of these measures across the 36 trials (Semrau et al., 2013).

For behavioral analyses, all eight robotic parameters were used (mean and variability measures). We initially ran the lesion analyses with all eight parameters, but found significant correlation between mean and variability on a given parameter, and significant overlap between mean and variability in the statistical lesion maps. Thus, only the four mean values of these measures are presented in the lesion analyses.

We transformed our two-sided ratio measures to one-sided measures, as required by our VLSM analysis, so that higher scores were indicative of worse performance. To do this, we used data from 74 healthy control subjects (mean age = 61, range 18–88 yr, 37 female, 64 right handed; Semrau et al., 2013) to identify the median and 95% range for control behavior. The following equation was used for ratio values greater than 1:

$$\begin{array}{l} \text{Transformed}\\ \text{score} \end{array}\text{​ }=\text{ ​}\frac{\left(\text{Subject Score}-\text{Control Median}\right)}{\left(97.5^{\text{th}}\text{ Control Percentile}-\text{Control Median}\right)}$$

The following equation was used for ratio values less than 1:

$$\begin{array}{l} \text{Transformed}\\ \text{score} \end{array}=\frac{\left(\text{Control Median}-\text{Subject Score}\right)}{\left(\text{Control Median}-2.5^{\text{th}}\text{ Control Percentile}\right)}$$

For all other parameters, normative ranges were calculated as the 95% range from control subjects. Stroke subjects who scored outside of the normative range on a given parameter were considered abnormal on that parameter. To classify overall task performance, those who were abnormal on greater than two parameters were considered to have failed the task overall since 95% of our controls were only abnormal on two or fewer parameters out of eight (i.e., subjects failed the task if they performed worse than 95% of healthy controls) (Semrau et al., 2013). This “pass/fail” classification was only used for behavioral analyses. All lesion analyses used the continuous measures from the robotic task.

### Image acquisition, lesion delineation, and normalization

Each subject underwent either MRI (*n* = 125) or noncontrast CT (*n* = 17) at the Foothills Medical Centre. T2-weighted fluid-attenuated inversion-recovery (FLAIR), diffusion weighted imaging (DWI), and apparent diffusion coefficient (ADC) sequences were performed according to the standard acute stroke MRI protocol at the Foothills Medical Centre. CT scans were also performed according to the standard acute stroke protocol at the Foothills Medical Centre. We specifically used CT images that were beyond the very acute stage post-stroke (mean = 2.1 days post-stroke), so we could better define the region of infarct, and only if an MRI was not done. If the region of infarct on CT was questionable or not well-defined according to a trained stroke neurologist, subjects were not included in our analyses. These radiological investigations were performed for clinical diagnostic purposes and obtained, with consent, for use in this study. Subjects with only CT scans were included in our analyses to increase our sample size and improve statistical power, as is commonly done in large VLSM studies (Karnath et al., 2009; Verdon et al., 2010; Winder et al., 2015). Those individuals where only a CT was performed typically had clear stroke symptoms and a well-defined area of infarct on CT. In our center, when there is a clear area of infarct on CT an MRI is often not done. Sperber and Karnath (2016) have argued that excluding subjects with only CT will likely inject bias into a sample in favor of smaller subcortical strokes. There is currently no consensus as to whether subjects with only CT imaging should be excluded from lesion analysis studies. Several studies have combined both CT and MR imaging (Bates et al., 2003; Karnath et al., 2004, 2011; Winder et al., 2015) while others use only MRI (Baier et al., 2010a,b; Meyer et al., 2016). We chose to include individuals with only CT. For MR imaging, either a Siemens or GE Medical Systems scanner at 1.5 or 3T was used, respectively. The in-plane resolution was consistent across all scan types at 1 mm^2^ and slice thickness varied from 3 to 5 mm depending on the scanner used with 0 mm interslice gaps.

Lesion location was marked by J.K. and/or S.F. directly on each axial slice of the T2-weighted FLAIR or non-contrast CT images using MRIcron software (Rorden et al., 2007) (http://www.mccauslandcenter.sc.edu/mricro/mricron/) to obtain a binary volume of interest (VOI) indicating the region of damaged tissue. The DWI and ADC images were used to accurately determine the location of acute brain damage caused by ischemia. Only areas of acute ischemia (hyperintense on DWI and hypointense on ADC) were included in the VOI. FLAIR hyperintensities with no corresponding DWI hyperintensity were not included in the lesion drawing, as these likely represented small microangiopathic changes or age-related white matter changes. In instances where a DWI hyperintensity was observed with little FLAIR hyperintensity (i.e., very acute scans), we marked the area of DWI hyperintensity on the FLAIR image. An experienced stroke neurologist (J.D. or A.Y.) who was blinded to subjects' clinical and robotic scores confirmed each lesion delineation and corrected markings where appropriate. Each subject's scan and VOI were then normalized to the Montreal Neurological Institute (MNI) template brain using the clinical toolbox in SPM8 (http://www.nitrc.org/projects/clinicaltbx) (Rorden et al., 2012; Winkler and Kochunov, 2012). CT images were transformed to MNI space using the “CT Normalization” function in the Clinical Toolbox. Images were first converted from Hounsfield units to the image brightness range of the template and then normalized using SPM8's standard normalization function. These images were then converted back to Hounsfield units (Rorden et al., 2012). MR images were transformed to MNI space using the “MR Normalization” function, since high-resolution T1 images were not available. SPM8's standard normalization procedure was then used, which first determines the optimal 12-parameter (translations, rotations, zooms, and shears) affine transformation followed by estimation of non-linear deformations (Ashburner and Friston, 1999). Default SPM8 settings were used primarily (spatial smoothing of anatomical image with 8 mm full width at half maximum (FWHM), smoothing of lesion with 3 mm FWHM and 0.5 threshold, affine regularization using the International Consortium for Brain Mapping (ICBM) space template, non-linear frequency cutoff: 25 mm, number of non-linear iterations: 16, non-linear regularization: 1, 1 × 1 × 1 mm voxel size, and automatic setting of origin). If the normalized images appeared distorted, we performed the normalization steps again, setting the origin manually. FLAIR and CT specific template images (in MNI space) were used for normalization. To prevent warping of damaged tissue during the normalization process, cost function masks were used for lesioned brain areas (Brett et al., 2001). Final normalized VOIs (binarized, warped, and smoothed) were compared to the original imaging to ensure accuracy, and used in subsequent imaging analyses.

### Voxel-based lesion symptom mapping and statistical region of interest analyses

Mean robotic task scores for each parameter were compared to lesion location using voxel-based lesion symptom mapping (Bates et al., 2003). At each voxel, robotic scores between the group with damage and the group without damage were compared using a *t*-test. This was performed using the non-parametric mapping (NPM) software available in the MRIcron software package. For response latency and initial direction error the absolute magnitude of error was used, with higher numbers indicating increasing impairment. For peak speed ratio and path length ratio, we used the one-sided transformed values as this provided a continuous one-sided measure of impairment on these parameters. To maintain sufficient statistical power only those voxels where a minimum of five subjects had damage were analyzed, which is a common threshold in VLSM studies (Kalénine et al., 2010; Lo et al., 2010; Geva et al., 2011; Molenberghs and Sale, 2011; Herbet et al., 2014). This threshold also ensures that we don't limit our “search area” to only the most common areas of damage. To correct for multiple comparisons we used a voxel-wise false discovery rate (FDR) correction (*q* = 0.01). Statistical maps are presented on the T1–weighted MNI template brain available in MRIcron.

For the region of interest analysis, 150 regions were defined. Cortical and subcortical regions were defined based on the Automated Anatomical Labeling Atlas (Tzourio-Mazoyer et al., 2002) and white matter tract regions were defined based on Neuroanatomy and Tractography Laboratory atlases (http://www.natbrainlab.com) (Catani and de Schotten, 2008). Specially designed software (Niistat; http://www.nitrc.org/projects/niistat) was operated in MATLAB (MathWorks, Natick, MA). For each region where at least five subjects had damage, the proportion of damage resulting from stroke and kinesthetic performance for those subjects were entered into a general linear model. This model tested whether the proportion of damage to a given region of interest was significantly associated with impaired kinesthesia. Results were converted to *z*-scores for each region and family-wise error was controlled via 4000 permutations (*p* < 0.05). This method improves statistical power by reducing the number of statistical tests performed.

### Clinical assessment

Standard clinical assessments were performed by a study physician or therapist with experience in stroke assessment. To assess proprioception of the stroke affected upper extremity the Thumb Localizing Test (TLT) was performed (Hirayama et al., 1999). During this test the subject's eyes were closed and their stroke affected arm was manipulated by the examiner and placed in space above eye level. The subject was then asked to use the thumb and forefinger of the ipsilesional hand to pinch the thumb of the hand that was being held in a static position by the therapist. Each subject was scored based on their ability to locate the thumb on a scale from 0 (no difficulty locating thumb) to 3 (unable to locate thumb).

A battery of other clinical assessments was performed within 1 day of the robotic assessment. An apraxia assessment (Zwinkels et al., 2004) was performed on individuals clinically suspected of having apraxia and they were excluded from the study. The Functional Independence Measure (FIM) (Keith et al., 1987) was used as an indicator of daily functional abilities. The Chedoke-McMaster Stroke Assessment (CMSA) Impairment Inventory for the upper extremities was completed to assess arm and hand motor function (Gowland et al., 1993). Finally, the Behavioral Inattention Test (BIT) provided an indication of visuospatial neglect in each subject (Wilson et al., 1987).

### Statistical analyses

Statistical comparisons in clinical, demographic and robotic outcomes were made between left and right hemisphere lesion subjects using independent samples *t*-tests or chi-squared tests when appropriate. Normality of our robotic parameters was tested using Kolmogorov-Smirnov tests. Analysis of covariance was used to account for lesion volume when identifying which variables significantly affected kinesthesia post-stroke. Spearman correlations were used to determine the relationship between demographic, clinical and robotic measurements. Statistical analyses were performed using IBM SPSS Statistics version 20. Corrections for multiple comparisons were conducted using a Bonferroni correction for behavioral data, a voxel-wise false discovery rate correction for voxel-based analyses and permutation testing for region of interest analyses.

## Results

### Subject demographics and clinical assessments

Subject demographics and clinical scores are presented in Table 1. The mean time between stroke onset and neuroimaging was 2.3 ± 2.8 days (MRI = 2.3 ± 2.8 days; CT = 2.1 ± 2.4 days) and the time between stroke and behavioral assessment was 8.9 ± 6.4 days. A total of 24 subjects were considered to have visuospatial neglect based on BIT scores of less than 130 (Halligan et al., 1991) (18 right hemisphere, 6 left hemisphere). Five subjects presented with involvement of more than one cerebral artery territory and thus are counted in all applicable categories in Table 1.

**Table 1:** **Subject demographics and clinical measures**.

	**Hemisphere of stroke**		
	**Left (*n* = 67)**	**Right (*n* = 75)**	**Total (*n* = 142)**
Age	60 ± 16	62 ± 4	61 ± 15
Sex	27F, 35M	20F, 48M	47F, 83M
Stroke Territory			
ACA/MCA/PCA	3/54/12	5/60/16	8/114/28
Lesion Vol. (mL)	20 ± 39.2	37.4 ± 50.0	23.1 ± 38.3
Handedness^*^: R/L/M	54/5/3	66/1/1	120/6/4
BIT	139 ± 12	132 ± 18	135 ± 16
TLT: 0/1/2/3	41/16/6/3^**^	40/23/8/4	81/39/15/7
CMSA^**^: 1/2/3/4/5/6/7			
Affected arm	4/1/6/5/10/13/27	6/4/5/5/16/12/27	10/5/11/10/26/25/54
Unaffected arm	0/0/0/0/0/0/66	0/0/0/0/0/0/75	0/0/0/0/0/0/141
FIM	106 ± 19	101 ± 23	106 ± 19

Values presented as mean ± standard deviation where applicable. F, Female; M, Male; ACA, Anterior Cerebral Artery; MCA, Middle Cerebral Artery; PCA, Posterior Cerebral Artery (Note: Six individuals had involvement of more than one territory and were counted in all applicable categories); R, Right; L, Left; M, Mixed; BIT, Behavioral Inattention Test; TLT, Thumb Localizing Test (0 = no impairment, 3 = marked impairment); CMSA, Chedoke McMaster Stroke Assessment for the upper extremity (7 = normal movement, 1 = flaccid paralysis). For TLT and CMSA, counts of the number of individuals within each score are reported. FIM, Functional Independence Measure.

* Handedness prior to stroke.

** TLT and CMSA scores unavailable for one subject.

Initially, we compared various clinical and demographic parameters of individuals with left and right hemisphere lesions. No significant differences were observed between groups in terms of age, FIM score (unpaired *t*-tests, *p* > 0.05), CMSA, or thumb localizing tests for the affected limb (Mann-Whitney *U*-tests, *p* > 0.05). On average, right hemisphere stroke subjects had significantly larger lesion volume (37.4 ± 50.0 mL) compared to left hemisphere stroke subjects (20 ± 39.2 mL; unpaired *t*-test, *p* = 0.005). Right hemisphere stroke subjects also had lower BIT scores (right = 132 ± 18 vs. left = 139 ± 12; unpaired *t*-test, *p* = 0.014).

### Robotic assessment of kinesthesia

Failure on the kinesthesia task, defined as falling outside of the normal healthy control range on three or more of the eight measured parameters (Semrau et al., 2013), occurred in 56% (*n* = 79) of subjects. Among right hemisphere strokes, 72% failed the task whereas 37% of left hemisphere subjects failed (Fisher's Exact test: *p* < 0.001). Thus, subjects with right hemisphere strokes had 1.93 times the risk of failing the kinesthesia task compared to left hemisphere strokes (risk ratio = 1.93; 95% confidence interval = 1.37–2.71).

Subjects with right hemisphere stroke had poorer performance overall on the kinesthesia task (**Table 3**). After correcting for multiple comparisons (*p* < 0.006, Bonferroni corrected, *n* = 8, *p* = 0.05) lesion volume was positively correlated with the total number of kinesthesia parameters failed (*r* = 0.39, *p* < 0.001). We also found that most of the individual kinesthesia parameters were correlated with lesion volume in our sample (Table 2). Correlations between individual parameters and clinical scores are shown in Table 2. Age did not correlate with performance on any robotic parameters or clinical scores (*p* > 0.05). Using analysis of covariance, including lesion volume (mL) as a covariate, and comparing left hemisphere to right hemisphere stroke subjects on each robotic parameter, we found that right hemisphere stroke subjects still performed worse compared to left hemisphere stroke subjects on the kinesthesia task (Table 3).

**Table 2:** **Spearman correlations between individual kinesthesia parameters and clinical assessments**.

	**RLv**	**PSR**	**PSRv**	**IDE**	**IDEv**	**PLR**	**PLRv**	**BIT**	**FIM**	**TLT**	**Vol.**
RL	0.756^‡^	0.233^†^	0.294^‡^	0.445^‡^	0.508^‡^	0.164	0.364^‡^	−0.431^‡^	−0.325^‡^	0.246^†^	0.328^‡^
RLv		0.258^†^	0.262^†^	0.538^‡^	0.559^‡^	0.138	0.447^‡^	−0.478^‡^	−0.347^‡^	0.355^‡^	0.340^‡^
PSR			−0.029	0.312^‡^	0.288^‡^	0.353^‡^	0.315^‡^	−0.267^‡^	−0.318^‡^	0.334^‡^	0.168^*^
PSRv				0.363^‡^	0.382^‡^	0.275^‡^	0.625^‡^	−0.133	−0.249^†^	−0.008	0.107
IDE					0.919^‡^	0.355^‡^	0.688^‡^	−0.484^‡^	−0.397^‡^	0.426^‡^	0.438^‡^
IDEv						0.306^‡^	0.655^‡^	−0.495^‡^	−0.379^‡^	0.407^‡^	0.401^‡^
PLR							0.453^‡^	−0.181^*^	−0.296^†^	0.183^*^	0.099
PLRv								−0.388^‡^	−0.450^‡^	0.298	0.297^†^
BIT									0.393^‡^	−0.278^†^	−0.517^‡^
FIM										−0.360^‡^	−0.237^†^
TLT											0.110

RL, response latency; RLv, response latency variability; PSR, peak speed ratio; PSRv, peak speed ratio variability; IDE, initial direction error; IDEv, initial direction error variability; PLR, path length ratio; PLRv, path length ratio variability; BIT, behavioral inattention test; FIM, functional independence measure; TLT, thumb localizing test. Vol: Lesion Volume.

* Significant at p < 0.05

† Significant at p < 001

‡ Significant at p < 0.001.

**Table 3:** **Analysis of Covariance between right and left hemisphere stroke subjects**.

**Parameter**	**Mean (L, R)**		***F*-value**	***P*-value**
	**Original**	**Adjusted**		
RL	0.55, 0.74	0.58, 0.72	8.03	0.055
RLv	0.28, 0.41	0.30, 0.40	9.49	0.064
PSR	0.61, 0.80	0.62, 0.80	2.93	0.089
PSRv	0.34, 0.39	0.35, 0.38	1.62	0.205
IDE	25.4, 36.2	27.1, 34.6	6.03	0.015
IDEv	20.7, 28.1	21.8, 27.1	6.45	0.012
PLR	0.85, 1.02	0.91, 0.97	0.16	0.686
PLRv	0.30, 0.40	0.33, 0.37	1.17	0.281
Total	2.28, 3.86	2.47, 3.70	10.97	0.001^*^

Performance on each parameter of the kinesthesia task was compared between left and right hemisphere stroke subjects. Lesion volume (mL) has been considered as a covariate. Original and adjusted mean values across 36 trials of the task are presented, with higher mean values indicating worse performance. Total, total number of kinesthesia parameters failed; L, Left; R, Right;

* indicates statistically significant difference (p < 0.006, Bonferroni corrected).

### Voxel-based lesion symptom mapping analysis

**Figure 2:**
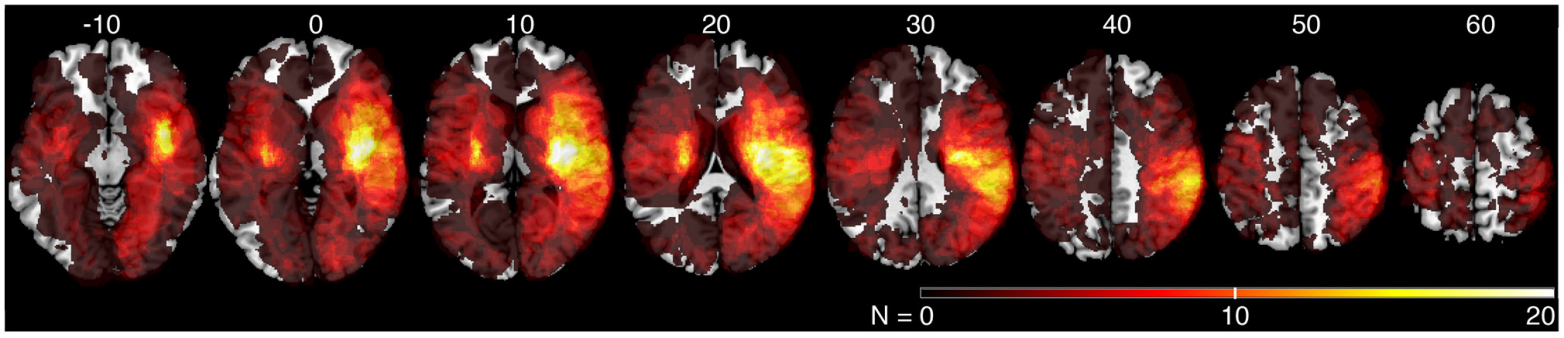
**Lesion overlap of all stroke subjects (***N*** = 142)**. MNI z-coordinates are presented above their respective axial slices. Color bar indicates the number of subjects with lesions at individual voxels. Brighter voxels indicate a greater number of overlapping lesions.

**Figure 3:**
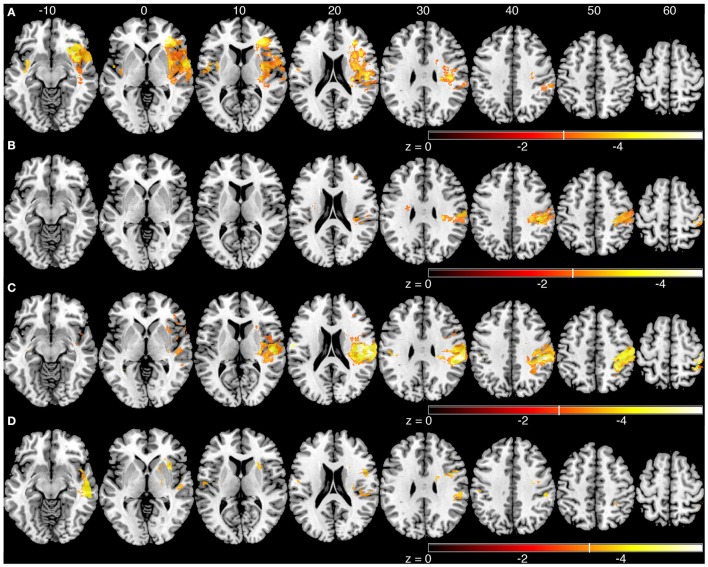
**Voxel-based analyses of mean parameters from the kinesthesia task**. Results from left and right hemisphere lesions are presented on the same template brain. Voxels that surpassed correction for multiple comparisons (*q* = 0.01, false discovery rate (FDR) corrected) are color coded. Brighter colors indicate voxels of increasing significance. White lines on each color bar indicate the FDR thresholds for each parameter. MNI z-coordinates are presented above their respective axial slices. **(A)** Voxels associated with increased response latency (threshold, left: *z* < −3.22, *q* = 0.01 FDR; right: *z* < −2.81, *q* = 0.01 FDR). **(B)** Voxels associated with poor peak speed ratio (threshold, left, and right: *z* < −2.33, *p* < 0.01, uncorrected). **(C)** Voxel associated with increased initial direction error (threshold, left: *z* < −3.55, *q* = 0.01 FDR; right: *z* < −2.88, *q* = 0.01 FDR). **(D)**. Voxels associated with poor path length ratio (threshold, left: z < −3.31, *q* = 0.05 FDR; right: *z* < −3.26, *q* = 0.01 FDR).

We generated an overlap map of all individual lesion locations as an initial indicator of lesion distribution (Figure 2). There was greater overlap of lesions in the right hemisphere. Little overlap was observed in left inferior and posterior parietal areas. Since there were differences in lesion distribution between left and right hemisphere stroke subjects, subsequent analyses were performed on left and right hemisphere lesions separately. Results of the VLSM analysis for the mean values from the kinesthesia task are shown in Figure 3, Table 4. The areas of brain damage associated with increased response latency (Figure 3A) are distributed through the right inferior frontal gyrus, inferior post-central gyri, and bilateral insula and frontal parietal operculum. Areas of damage associated with poor performance on peak speed ratio (Figure 3B) involve a much smaller area centered on the right post-central and supramarginal gyri as well as the left frontal subcortical white matter. Initial direction error (Figure 3C) was associated with lesions to bilateral post-central gyri and right supramarginal gyrus, angular gyrus, superior temporal lobe and the posterior aspect of the insula. Errors in path length ratio (Figure 3D), a measure of movement amplitude perception, were associated with lesions to the right superior and middle temporal gyri, anterior insula and bilateral parietal operculum, pre-central gyrus and supramarginal gyrus. The peak speed ratio parameter did not survive the stringent false discovery rate correction and so is presented at a more liberal threshold of *p* < 0.01, uncorrected.

**Table 4:** **Coordinates of peak activation from voxel-based lesion symptom mapping analyses (Montreal Neurological Institute coordinates)**.

**Parameter**	**Coordinates**			**Cluster Size (# voxels)**	**Max *z*-score**
	**x**	**y**	**z**		
**RESPONSE LATENCY**					
	39	11	−16	60091	−5.57
	−49	−12	11	1533	−4.30
	−35	1	−16	1175	−4.21
	−48	−2	−2	166	−3.77
	40	−26	−9	184	−3.62
	−55	−3	25	166	−3.59
	−50	−1	33	57	−3.59
	−37	14	−7	50	−3.44
	38	−36	31	21	−3.25
	−31	−7	−17	13	−3.23
	−41	10	−8	43	−3.22
	59	8	16	143	−3.21
	60	−35	15	51	−3.11
**PEAK SPEED RATIO**					
	45	−28	39	15875	−4.11
	−23	−6	27	474	−3.71
	17	19	3	100	−3.13
	32	−33	19	28	−2.89
	32	37	18	38	−2.69
	51	−16	22	30	−2.65
	60	−40	37	20	−2.60
	44	−17	26	14	−2.58
	−28	−40	51	13	−2.45
**INITIAL DIRECTION ERROR**					
	46	−22	19	47560	−6.41
	−63	−11	13	271	−4.72
	−47	−21	31	719	−4.72
	−36	−43	2	41	−4.19
	29	18	13	168	−3.87
	27	−26	51	22	−3.85
	38	3	32	97	−3.76
	35	13	−4	390	−3.71
	−18	−26	10	42	−3.65
	44	28	33	14	−3.61
	51	7	24	309	−3.58
	−36	−19	−13	15	−3.57
	−41	−25	25	56	−3.56
	50	24	−4	293	−3.39
	32	6	33	21	−3.32
	50	−4	−12	30	−3.30
	50	5	−8	84	−3.30
	59	14	27	13	−3.27
	49	−51	1	27	−3.15
	41	43	12	10	−3.12
	60	14	−2	21	−3.09
	62	9	−1	10	−3.09
	39	−9	−10	16	−3.09
	56	−41	1	39	−3.07
	40	−74	42	140	−3.04
	32	−58	50	53	−3.01
	22	6	20	10	−3.01
	38	−11	−5	14	−2.99
	59	8	16	16	−2.98
	43	−69	11	26	−2.95
	34	39	9	56	−2.93
**PATH LENGTH RATIO**					
	50	−5	35	1757	−5.57
	27	13	9	1333	−4.93
	−31	−6	14	19	−4.68
	62	−28	−8	6448	−4.61
	38	−49	53	16	−4.36
	37	−34	33	27	−4.36
	22	−4	−3	164	−4.34
	18	11	2	337	−4.34
	20	−2	33	294	−4.33
	48	−29	39	2332	−4.24
	29	−1	−17	65	−4.12
	44	28	33	13	−3.90
	23	13	20	44	−3.87
	31	15	−18	100	−3.78
	50	−1	35	32	−3.64
	31	−19	17	133	−3.59
	29	−24	21	18	−3.53
	57	−17	15	12	−3.51
	−52	−10	8	395	−3.50
	−30	−28	36	11	−3.50
	−52	−23	31	14	−3.48
	−43	−29	34	52	−3.48
	−51	−24	37	48	−3.48
	−42	−31	41	59	−3.48
	14	−9	25	93	−3.47
	32	6	2	11	−3.43
	22	10	−2	60	−3.37
	33	−45	45	120	−3.35
	34	32	21	10	−3.34
	43	−19	16	27	−3.30
	21	0	−9	25	−3.26

A comparison of three of the kinesthetic parameters (peak speed ratio, initial direction error, and path length ratio) are presented in **Figure 5**. The response latency parameter was not included in this figure since this parameter was simply the time taken to respond to the passive movement. The other three parameters measure the kinematics of the movement (speed, direction, amplitude). Impairments in all three parameters were associated with damage to the supramarginal gyrus in the right hemisphere.

### Statistical region of interest analysis

**Figure 4:**
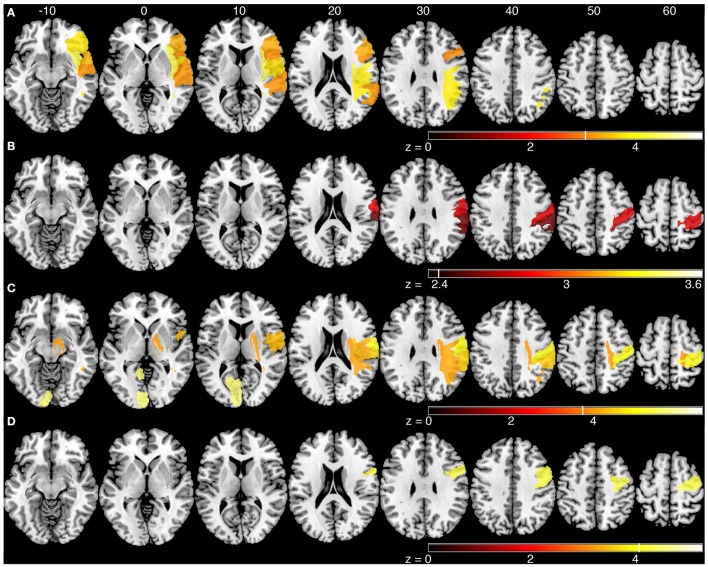
**Statistical region of interest analyses for mean parameters from the kinesthesia task**. Regions that surpassed correction for multiple comparisons (*p* = 0.05, 4000 permutations) are shown, and are indicated by the vertical white line on each color bar. MNI z-coordinates are presented above their respective axial slices. Color bars indicate *z*-scores from the general linear model, with brighter colors indicating greater *z*-scores. **(A)** Regions associated with increased response latency (threshold, *z* > 3.04, 4000 permutations). **(B)** Regions associated with poor peak speed ratio (threshold, *z* > 2.33; *p* < 0.01, uncorrected). **(C)** Regions associated with increased initial direction error (threshold, left: *z* > 5.60, 4000 permutations; right: *z* > 3.83, 4000 permutations). **(D)** Regions associated with poor path length ratio (threshold, *z* > 4.07, 4000 permutations).

The sROI analysis examined whether the proportion of damage to each region was associated with poor performance on the robotic task. Several regions were associated with poor response latency that were highly distributed throughout the MCA territory, including the right inferior frontal gyrus, insula, superior temporal gyrus, and arcuate fasciculus (Figure 4A, Table 5). Impaired peak speed ratio was not associated with proportion of damage in any region after correction for multiple comparisons (*p* < 0.05, 4000 permutations). These results are presented at *p* < 0.01, uncorrected (Figure 4B). Impaired initial direction error was significantly associated with damage to the right post-central, superior and inferior parietal, and supramarginal gyri (Figure 4C). Proportion of damage to the left calcarine sulcus was associated with initial direction errors. Interestingly, impaired path length ratio was associated with damage to the right pre-central gyrus (Figure 4D). The individual regions and *z*-scores are presented in Table 5. There was good agreement between the VLSM and sROI techniques that we employed.

**Table 5:** **Regions where increased damage was associated with increased kinesthetic impairment**.

**Parameter**	**Region**	***z*-score**
**RESPONSE LATENCY (RL)**		
	Insula	4.33
	Arcuate, anterior segment	4.23
	Arcuate, long segment	3.91
	Arcuate fasciculus	3.84
	Inferior frontal gyrus, orbital part	3.82
	Rolandic operculum	3.72
	Temporal pole, superior gyrus	3.46
	Inferior frontal gyrus, triangular part	3.34
	Superior temporal gyrus	3.16
	Inferior frontal operculum	3.09
**PEAK SPEED RATIO (PSR)**		
	0 “regions survived” threshold	
**INITIAL DIRECITON ERROR (IDE)**		
	Calcarine sulcus^*^	5.60
	Post-central gyrus	4.98
	Supramarginal gyrus	4.43
	Arcuate, anterior segment	4.37
	Rolandic operculum	4.24
	Arcuate fasciculus	4.00
	Corticospinal tract	3.88
**PATH LENGTH RATIO (PLR)**		
	Precentral gyrus	4.27

Regions of interest defined base on the Automated Anatomical Labeling Atlas and Catani Atlas. Only regions that surpassed the statistical threshold are presented.

* This region only is in the left hemisphere.

## Discussion

The main finding of our study is that impairments in kinesthesia related to lesions in several brain regions that were largely dissociable depending on the type of impairment (i.e., speed vs. amplitude perceptions). While most aspects of kinesthesia involved the post-central gyrus, several other brain areas including the supramarginal, angular, pre-central, and superior temporal gyri as well as the insula were relevant. These results suggest that a distributed network is involved in our kinesthetic awareness, with some specialization of processing within this network.

### Lesion correlates of kinesthetic perceptions

Longer response latency was associated primarily with lesions to the right inferior frontal gyrus, bilateral insula and parietal operculum (Figures 3A, 4A). Conversely, lesions in frontal and parietal cortices were more associated with the kinematics of movement perception. Among the various functions of the insula, it has been proposed as a structure that integrates somatosensory information to create a sense of body and self-awareness (Karnath, 2005). This body awareness processing likely informs us of our current body state and any changes to that state, such as the kinesthetic stimulus in our task. The insula is likely an important structure for identifying that a change in body state has occurred (i.e., passive arm movement) while structures in frontal and parietal cortices are important for identifying the specific nature and properties of that change (i.e., speed, direction, and amplitude of movement).

Difficulty in matching the speed of movement was significantly associated with lesions in the post-central and supramarginal gyri (Figures 3B, 4B). Previous research involving single cell recordings in non-human primates has shown that neurons in Brodmann's area 2 and 5 of the parietal lobe contain cells that modulate their activity in relation to the velocity of limb movement during motor tracing tasks (Averbeck et al., 2005). The ability of subjects to perceive the speed of passively generated limb movements, in order to guide movements of the ipsilesional limb, was significantly affected by lesions to the post-central gyrus. Directional errors were also linked to lesions along the post-central gyrus, in addition to lesions in the posterior parietal cortex, superior temporal lobe and insula (Figures 3C, 4C). Along with the post-central gyrus, it has been observed that neurons in primate Broadmann's area 5 respond to specific directions of movement during reaching (Kalaska et al., 1983). Accurate perception of movement direction may be a more complicated and integrative process than perception of movement speed, and thus requires more processing from these highly integrative areas such as the posterior parietal cortex and parietal operculum.

**Figure 5:**
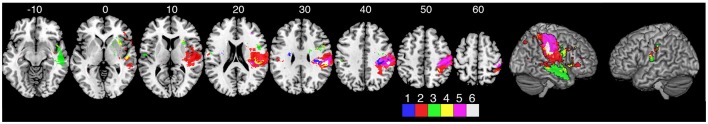
**Overlap analysis of three of the kinematic measures from the kinesthesia task**. The peak speed ratio parameter was not included in this figure since this parameter was simply the time taken to respond to the passive movement. The other three parameters measure the kinematics of the movement (speed, direction, amplitude). Each color represents an individual measure or overlap of kinematic measures from the VLSM analysis. (1) peak speed ratio, (2) initial direction error, (3) path length ratio, (4) path length ratio + initial direction error overlap, (5) peak speed ratio + initial direction error overlap, (6) all three parameters overlap.

Impairments in matching the amplitude of movement (PLR) were significantly associated with lesions to the right superior and middle temporal gyri, anterior insula, supramarginal gyrus, bilateral parietal opercula (Figure 3D) and pre-central gyrus (Figure 4D). It is interesting that the sROI analysis revealed that the amount of damage to the pre-central gyrus, and not the post-central gyrus, was a significant predictor of impairment in matching the amplitude of movement (Figure 4D). Previous functional MRI studies using passive movement stimuli in healthy controls have shown robust activations of both pre- and post-central gyri (Naito et al., 1999, 2007). Our results suggest that the amount of damage to the pre-central gyrus may be an important indicator for impaired perception of movement amplitude. With the VLSM analysis three of the kinematic parameters (peak speed Ratio, Initial Direction Error, Path Length Ratio) shared a common lesion location (right supramarginal gyrus) that was significantly associated with kinesthetic impairments (Figure 5, white area). The right supramarginal gyrus has recently been implicated in proprioceptive processing at the wrist using functional MRI in healthy controls and stroke subjects (Ben-Shabat et al., 2015).

It should be noted that our robotic task (mirror matching movements of the opposite limb without vision) is a complex behavior as it requires subjects to not only attend to the affected arm that is moved by the robot, but also execute a movement with their unaffected arm. Thus, it requires attentional, executive, and higher motor processing in addition to kinesthetic processing. These other processes may be a contributor to the distribution in lesion maps we observed. In particular, damage to the right superior and middle temporal gyri (as seen in our response latency and path length ratio parameters) and inferior parietal lobule have been associated with visuospatial neglect post-stroke (Karnath et al., 2011) and damage to left hemisphere frontal parietal areas have been associated with impaired executive function (Barbey et al., 2012).

### Hemispheric differences in kinesthetic processing?

Our analysis identified lesions in the right hemisphere that correlated with different behavioral parameters. In contrast, we were unable to find corresponding relationships for the left hemisphere. This difference between left and right hemispheres may be due to several factors. First, our behavioral results showed that right hemisphere stroke subjects failed more parameters overall on the kinesthesia task and thus provide a greater range of impairments for comparing to lesion location (Table 3). Second, there were significant differences in lesion locations and lesion volumes between left and right hemisphere subjects in our sample (Table 1, Figure 2). Critically, we limited recruitment of many left hemisphere cortical lesions due to aphasia (Bates et al., 2003), as it was important that they understood the instructions for the kinesthesia task. We suspect that unfortunately, we simply had an insufficient overlap of lesions to accurately map where kinesthetic processing occurs in the left hemisphere, or to determine whether any lateralization in kinesthetic processing exists in the present study.

Some evidence has suggested right hemisphere dominance in processing proprioceptive information in right-handers. Goble and Brown (2010) have demonstrated that healthy subjects performed better on a dynamic position matching task in the upper extremities when matching to their non-dominant arm, suggesting a right hemisphere specialization for proprioceptive feedback processing. Additionally, the right hemisphere has been proposed to play a specialized role in sensorimotor stabilization mechanisms for accurately reaching goal targets (Mutha et al., 2012). Further research is needed to determine the potential lateralization of kinesthetic processing, and its relationship to sensorimotor function.

## Limitations

One of the challenges in working with patients so early after stroke is that many have language deficits that prohibit participation, and this limits our ability to comment on left hemisphere involvement. The technique of VLSM also does not account for diaschesis effects (dysfunction of undamaged brain structures due to disconnections), or the fact that vascular lesions follow characteristic patterns, which can potentially introduce systematic biases in the results (Mah et al., 2014). A further consideration is that the present study only examined the impact of cerebral lesions on kinesthetic awareness. Future research should consider cerebellar involvement as well, given the known proprioceptive projections from the limbs to cerebellum (Manto et al., 2012). Combining both CT and MR imaging into our analyses is also controversial, as CT does not offer the same spatial resolution as MRI. Additionally, systematically including or excluding subjects with only CT may introduce bias in the results in favor of larger or smaller strokes, respectively (Sperber and Karnath, 2016). The present lesion analysis does provide insight into the distributed nature of kinesthetic processing. However, other methodologies such as functional MRI may provide a complementary approach to understanding the central processing of kinesthesia.

## Conclusions

We performed voxel-based lesion symptom mapping and statistical region of interest analyses on 142 sub-acute ischemic stroke subjects to assess which lesion locations were significantly associated with kinesthetic impairment on a robotic measure. We observed that a variety of lesion locations were associated with impairments in kinesthesia following stroke and that the type of impairment (ie. speed, direction, or amplitude of movement) is likely related to specific lesion locations. In addition to the separation in lesion locations, there was a common lesion location that was significantly associated with kinesthetic impairment, the right supramarginal gyrus. These results further our understanding of the human brain structures involved in kinesthetic processing and may help identify stroke survivors likely to have kinesthetic impairments based on lesion location.

## Author contributions

JK was responsible for project design, patient recruitment, data analysis, and writing of the manuscript. JS and SF assisted with project design, data analysis, and writing of manuscript. AY and JD provided expert opinion on lesion markings and editing of manuscript. TH and SS assisted with data analysis and writing of manuscript. MH assisted with project design and writing of the manuscript. SD is the primary investigator for the RESTART cohort and assisted with project design, patient recruitment, data analysis and writing of manuscript.

### Conflict of interest statement

SS is cofounder and chief scientific officer of BKIN Technologies, the company that commercializes the KINARM robotic device used in this study. The other authors declare that the research was conducted in the absence of any commercial or financial relationships that could be construed as a potential conflict of interest.

## References

[B1] Ashburner J. Friston K. J. (1999). Non-linear spatial normalization using basis functions. Hum. Brain Mapp. 7, 254–266. 10408769 10.1002/(SICI)1097-0193(1999)7:4<254::AID-HBM4>3.0.CO;2-G PMC6873340

[B2] Averbeck B. B. Chafee M. V. Crowe D. A. Georgopoulos A. P. Bruno B. Chafee M. V.. (2005). Parietal representation of hand velocity in a copy task. J. Neurophysiol. 93, 508–518. 10.1152/jn.00357.2004 15269226

[B3] Baier B. de Haan B. Mueller N. Thoemke F. Birklein F. Dieterich M.. (2010a). Anatomical correlate of positive spontaneous visual phenomena, A voxelwise lesion study. Neurology 74, 218–222. 10.1212/WNL.0b013e3181cb3e64 20083797

[B4] Baier B. Mueller N. Fechir M. Dieterich M. (2010b). Line bisection error and its anatomic correlate. Stroke 41, 1561–1563. 10.1161/STROKEAHA.109.576298 20489175

[B5] Barbey A. K. Colom R. Solomon J. Krueger F. Forbes C. Grafman J. (2012). An integrative architecture for general intelligence and executive function revealed by lesion mapping. Brain 135, 1154–1164. 10.1093/brain/aws021 22396393 PMC3326251

[B6] Bates E. Wilson S. M. Saygin A. P. Dick F. Sereno M. I. Knight R. T.. (2003). Voxel-based lesion-symptom mapping. Nat. Neurosci. 6, 448–450. 10.1038/nn1050 12704393

[B7] Ben-Shabat E. Matyas T. A. Pell G. S. Brodtmann A. Carey L. M. (2015). The right supramarginal gyrus is important for proprioception in healthy and stroke-affected participants: a functional MRI Study. Front. Neurol. 6:248. 10.3389/fneur.2015.00248 26696951 PMC4668288

[B8] Brett M. Leff A. P. Rorden C. Ashburner J. (2001). Spatial normalization of brain images with focal lesions using cost function masking. Neuroimage 14, 486–500. 10.1006/nimg.2001.0845 11467921

[B9] Catani M. de Schotten M. T. (2008). A diffusion tensor imaging tractography atlas for virtual *in vivo* dissections. Cortex 44, 1105–1132. 10.1016/j.cortex.2008.05.004 18619589

[B10] Geva S. Jones P. S. Crinion J. T. Price C. J. Baron J.-C. Warburton E. A. (2011). The neural correlates of inner speech defined by voxel-based lesion-symptom mapping. Brain 134, 3071–3082. 10.1093/brain/awr232 21975590 PMC3187541

[B11] Goble D. J. Brown S. H. (2010). Upper limb asymmetries in the perception of proprioceptively determined dynamic position sense. J. Exp. Psychol. Hum. Percept. Perform. 36, 768–775. 10.1037/a0018392 20515203

[B12] Goble D. J. Coxon J. P. Van Impe A. Geurts M. Van Hecke W. Sunaert S.. (2012). The neural basis of central proprioceptive processing in older versus younger adults: an important sensory role for right putamen. Hum. Brain Mapp. 33, 895–908. 10.1002/hbm.21257 21432946 PMC6870471

[B13] Goodwin G. M. Mccloskey D. I. Matthews P. B. C. (1972). The contribution of muscle afferents to kinesthesia shown by vibration induced illusions of movement and by the effects of paralysing joint afferents. Brain 95, 705–748. 10.1093/brain/95.4.705 4265060

[B14] Gowland C. Stratford P. Ward M. Moreland J. Torresin W. Van Hullenaar S.. (1993). Measuring physical impairment and disability with the Chedoke-McMaster Stroke Assessment. Stroke 24, 58–63. 10.1161/01.STR.24.1.58 8418551

[B15] Halligan P. W. Cockburn J. Wilson B. A. (1991). The behavioural assessment of visual neglect. Neuropsychol. Rehabil. 1, 5–32. 10.1080/09602019108401377

[B16] Herbet G. Lafargue G. Bonnetblanc F. Moritz-Gasser S. Menjot de Champfleur N. Duffau H. (2014). Inferring a dual-stream model of mentalizing from associative white matter fibres disconnection. Brain 137, 944–959. 10.1093/brain/awt370 24519980

[B17] Hirayama K. Fukutake T. Kawamura M. (1999). “Thumb localizing test” for detecting a lesion in the posterior column-medial lemniscal system. J. Neurol. Sci. 167, 45–49. 10.1016/s0022-510x(99)00136-7 10500261

[B18] Kalaska J. F. Caminiti R. Georgopoulos A. P. (1983). Cortical mechanisms related to the direction of two-dimentional arm movements: relations in parietal area 5 and comparison with motor cortex. Exp. Brain Res. 51, 247–260. 10.1007/BF00237200 6617794

[B19] Kalénine S. Buxbaum L. J. Coslett H. B. (2010). Critical brain regions for action recognition: lesion symptom mapping in left hemisphere stroke. Brain 133, 3269–3280. 10.1093/brain/awq210 20805101 PMC2965423

[B20] Karnath H. (2005). Awareness of the functioning of one's own limbs mediated by the insular cortex? J. Neurosci. 25, 7134–7138. 10.1523/JNEUROSCI.1590-05.2005 16079395 PMC6725240

[B21] Karnath H.-O. Fruhmann Berger M. Küker W. Rorden C. (2004). The anatomy of spatial neglect based on voxelwise statistical analysis: a study of 140 patients. Cereb. Cortex 14, 1164–1172. 10.1093/cercor/bhh076 15142954

[B22] Karnath H.-O. Rennig J. Johannsen L. Rorden C. (2011). The anatomy underlying acute versus chronic spatial neglect: a longitudinal study. Brain 134, 903–912. 10.1093/brain/awq355 21156661 PMC3044829

[B23] Karnath H.-O. Rorden C. Ticini L. F. (2009). Damage to white matter fiber tracts in acute spatial neglect. Cereb. Cortex 19, 2331–2337. 10.1093/cercor/bhn250 19168667 PMC2742593

[B24] Kavounoudias A. Roll J. P. Anton J. L. Nazarian B. Roth M. Roll R. (2008). Proprio-tactile integration for kinesthetic perception: an fMRI study. Neuropsychologia 46, 567–575. 10.1016/j.neuropsychologia.2007.10.002 18023825

[B25] Keith R. A. Granger C. V. Hamilton B. B. Sherwin F. S. (1987). The functional independence measure: a new tool for rehabilitation. Adv. Clin. Rehabil. 1, 6–18. 3503663

[B26] Kenzie J. M. Semrau J. A. Findlater S. E. Herter T. M. Hill M. D. Scott S. H.. (2014). Anatomical correlates of proprioceptive impairments following acute stroke: a case series. J. Neurol. Sci. 342, 52–61. 10.1016/j.jns.2014.04.025 24819922

[B27] Kim J. S. (1992). Pure sensory stroke. Clinical-radiological correlates of 21 cases. Stroke 23, 983–987. 10.1161/01.STR.23.7.983 1615549

[B28] Kim J. S. (2007). Patterns of sensory abnormality in cortical stroke: evidence for a dichotomized sensory system. Neurology 68, 174–180. 10.1212/01.wnl.0000251298.12763.9b 17224568

[B29] Lo R. Gitelman D. Levy R. Hulvershorn J. Parrish T. (2010). Identification of critical areas for motor function recovery in chronic stroke subjects using voxel-based lesion symptom mapping. Neuroimage 49, 9–18. 10.1016/j.neuroimage.2009.08.044 19716427

[B30] Mah Y. H. Husain M. Rees G. Nachev P. (2014). Human brain lesion-deficit inference remapped. Brain 137, 2522–2531. 10.1093/brain/awu164 24974384 PMC4132645

[B31] Manto M. Bower J. M. Conforto A. B. Delgado-García J. M. da Guarda S. N. F. Gerwig M.. (2012). Consensus paper: roles of the cerebellum in motor control–the diversity of ideas on cerebellar involvement in movement. Cerebellum 11, 457–487. 10.1007/s12311-011-0331-9 22161499 PMC4347949

[B32] Meyer S. Kessner S. S. Cheng B. Bönstrup M. Schulz R. Hummel F. C.. (2016). Voxel-based lesion-symptom mapping of stroke lesions underlying somatosensory deficits. NeuroImage Clin. 10, 257–266. 10.1016/j.nicl.2015.12.005 26900565 PMC4724038

[B33] Mima T. Sadato N. Yazawa S. Hanakawa T. Fukuyama H. Yonekura Y.. (1999). Brain structures related to active and passive finger movements in man. Brain 122(Pt 1),1989–1997. 10.1093/brain/122.10.1989 10506099

[B34] Molenberghs P. Sale M. V. (2011). Testing for spatial neglect with line bisection and target cancellation: are both tasks really unrelated? PLoS One 6:e23017. 10.1371/journal.pone.0023017 21829578 PMC3145773

[B35] Mutha P. K. Haaland K. Y. Sainburg R. L. (2012). The effects of brain lateralization on motor control and adaptation. J. Mot. Behav. 44, 455–469. 10.1080/00222895.2012.747482 23237468 PMC3549328

[B36] Naito E. Ehrsson H. H. Geyer S. Zilles K. Roland P. E. (1999). Illusory arm movements activate cortical motor areas: a positron emission tomography study. J. Neurosci. 19, 6134–6144. 10407049 10.1523/JNEUROSCI.19-14-06134.1999 PMC6783063

[B37] Naito E. Nakashima T. Kito T. Aramaki Y. Okada T. Sadato N. (2007). Human limb-specific and non-limb-specific brain representations during kinesthetic illusory movements of the upper and lower extremities. Eur. J. Neurosci. 25, 3476–3487. 10.1111/j.1460-9568.2007.05587.x 17553017

[B38] Proske U. Gandevia S. C. (2012). The proprioceptive senses: their roles in signaling body shape, body position and movement, and muscle force. Physiol. Rev. 92, 1651–1697. 10.1152/physrev.00048.2011 23073629

[B39] Romaiguère P. Anton J. Roth M. Casini L. Roll J. (2003). Motor and parietal cortical areas both underlie kinaesthesia. Cogn. Brain Res. 16, 74–82. 10.1016/S0926-6410(02)00221-5 12589891

[B40] Rorden C. Bonilha L. Fridriksson J. Bender B. Karnath H. O. (2012). Age-specific CT and MRI templates for spatial normalization. Neuroimage 61, 957–965. 10.1016/j.neuroimage.2012.03.020 22440645 PMC3376197

[B41] Rorden C. Karnath H.-O. Bonilha L. (2007). Improving lesion-symptom mapping. J. Cogn. Neurosci. 19, 1081–1088. 10.1162/jocn.2007.19.7.1081 17583985

[B42] Semrau J. A. Herter T. M. Scott S. H. Dukelow S. P. (2013). Robotic identification of kinesthetic deficits after stroke. Stroke 44, 3414–3421. 10.1161/strokeaha.113.002058 24193800

[B43] Sherrington C. (1907). On the proprioceptive system, especially in its reflex aspect. Brain 29, 467–485. 10.1093/brain/29.4.467

[B44] Sperber C. Karnath H. O. (2016). Topography of acute stroke in a sample of 439 right brain damaged patients. NeuroImage Clin. 10, 124–128. 10.1016/j.nicl.2015.11.012 26759787 PMC4683427

[B45] Tong D.-M. Zhou Y.-T. Wang G.-S. Cheng X.-D. Yang T.-H. Chang C.-H.. (2010). Hemorrhagic pure sensory stroke in the thalamus and striatocapsular area: causes, clinical features and long-term outcome. Eur. Neurol. 64, 275–279. 10.1159/000320938 20978367

[B46] Torre K. Hammami N. Metrot J. van Dokkum L. Coroian F. Mottet D.. (2013). Somatosensory-related limitation for bimanual coordination after stroke. Neurorehabil. Neural Repair 27, 507–515. 10.1177/1545968313478483 23474542

[B47] Tzourio-Mazoyer N. Landeau B. Papathanassiou D. Crivello F. Etard O. Delcroix N.. (2002). Automated anatomical labeling of activations in SPM using a macroscopic anatomical parcellation of the MNI MRI single-subject brain. Neuroimage 15, 273–289. 10.1006/nimg.2001.0978 11771995

[B48] Verdon V. Schwartz S. Lovblad K.-O. Hauert C.-A. Vuilleumier P. (2010). Neuroanatomy of hemispatial neglect and its functional components: a study using voxel-based lesion-symptom mapping. Brain 133, 880–894. 10.1093/brain/awp305 20028714

[B49] Weiller C. Jüptner M. Fellows S. Rijntjes M. Leonhardt G. Kiebel S.. (1996). Brain representation of active and passive movements. Neuroimage 4, 105–110. 9345502 10.1006/nimg.1996.0034

[B50] Wilson B. Cockburn J. Halligan P. (1987). Development of a behavioral test of visuospatial neglect. Arch. Phys. Med. Rehabil. 68, 98–102. 3813864

[B51] Winder K. Seifert F. Ohnemus T. Sauer E.-M. Kloska S. Dörfler A.. (2015). Neuroanatomic correlates of post-stroke hyperglycemia. Ann. Neurol. 77, 262–268. 10.1002/ana.24322 25448374

[B52] Winkler A. M. Kochunov P. G. D. (2012). FLAIR Templates. Available online at: http://glahngroup.org

[B53] Zwinkels A. Geusgens C. van de Sande P. Van Heugten C. (2004). Assessment of apraxia: inter-rater reliability of a new apraxia test, association between apraxia and other cognitive deficits and prevalence of apraxia in a rehabilitation setting. Clin. Rehabil. 18, 819–827. 10.1191/0269215504cr816oa 15573839

